# High-Temperature Raman Spectroscopy of Nano-Crystalline Carbon in Silicon Oxycarbide

**DOI:** 10.3390/ma11010093

**Published:** 2018-01-09

**Authors:** Felix Rosenburg, Emanuel Ionescu, Norbert Nicoloso, Ralf Riedel

**Affiliations:** Institut für Material- und Geowissenschaften, Technische Universität Darmstadt, Otto-Berndt-Straße 3, 64287 Darmstadt, Germany; ionescu@materials.tu-darmstadt.de (E.I.); nn@eurad.com (N.N.); riedel@materials.tu-darmstadt.de (R.R.)

**Keywords:** polymer-derived ceramics, Raman spectroscopy, anharmonicity, carbon, defects

## Abstract

The microstructure of segregated carbon in silicon oxycarbide (SiOC), hot-pressed at *T* = 1600 °C and *p* = 50 MPa, has been investigated by VIS Raman spectroscopy (λ = 514 nm) within the temperature range 25–1000 °C in air. The occurrence of the G, D’ and D bands at 1590, 1620 and 1350 cm^−1^, together with a lateral crystal size *L_a_* < 10 nm and an average distance between lattice defects *L_D_* ≈ 8 nm, provides evidence that carbon exists as nano-crystalline phase in SiOC containing 11 and 17 vol % carbon. Both samples show a linear red shift of the G band up to the highest temperature applied, which is in agreement with the description of the anharmonic contribution to the lattice potential by the modified Tersoff potential. The temperature coefficient χ_G_ = −0.024 ± 0.001 cm^−1^/°C is close to that of disordered carbon, e.g., carbon nanowalls or commercial activated graphite. The line width of the G band is independent of temperature with FWHM-values of 35 cm^−1^ (C-11) and 45 cm^−1^ (C-17), suggesting that scattering with defects and impurities outweighs the phonon-phonon and phonon-electron interactions. Analysis of the Raman line intensities indicates vacancies as dominating defects.

## 1. Introduction

Phonons are of fundamental importance for crystalline materials. The analysis of Raman active modes is well-suited to assessing the lattice dynamics of, e.g., carbon and its allotropes graphite [1,2], carbon nanotubes, [3,4,5] graphene, [6,7] amorphous carbon, [8,9] and diamond-like carbon [10,11]. It is very sensitive to the hybridization state of carbon, and provides information about the microstructure and properties such as the thermal conductivity, the specific heat and the thermal expansion [12].

In this work, we present a temperature-dependent Raman study of silicon oxycarbide (SiOC) containing 11 and 17 vol % of segregated carbon (labeled as C-11 and C-17 in the following). Amorphous SiO_2_ and fine dispersed carbon are the main constituents of these ceramic nano-composites [13]. Due to its unique microstructure, SiOC possesses a variety of interesting properties, such as high temperature and corrosion stability [14,15,16], high creep resistance [17] and high piezoresistivity [18,19]. Furthermore, SiOC-based materials have been shown to exhibit promising behavior as anode materials in Li-ion batteries [20]. Interestingly, the mentioned properties seem to be determined by the segregated carbon phase randomly distributed within the SiOC matrix. Depending on the concentration of carbon and the synthesis conditions, different carbon structures can be observed, as shown, e.g., in ref. [21]. At low concentrations and low pyrolysis temperatures (T < 1000 °C), highly disordered carbon domains embedded in an insulating SiOC matrix exist. At volume fractions larger than ≈5 vol % and synthesis temperatures beyond 1400 °C, a randomly grown continuous carbon network is formed [22]. Accordingly, C-11 and C-17 represent samples with concentrations well beyond the percolation threshold, ensuring that their properties solely depend on the state of the carbon within the composite material.

As mentioned above, the segregated carbon in silicon oxycarbide-based materials seems to determine many of its properties. As some of those are present or may be used at high temperatures (e.g., near-zero creep or piezoresistive behavior), it seems appropriate to study the nature and the temperature-dependent evolution of the segregated carbon phase in SiOC materials at high temperatures. Thus, the aim of the present study was to investigate the temperature evolution of the segregated carbon in SiOC by using Raman spectroscopy.

At ambient pressure, the thermal investigation of carbon is restricted to temperatures up to about 600 °C where carbon starts to oxidize. The segregated carbon in C-11 and C-17, on the contrary, is covered by silica, which protects it from oxidation, thus allowing investigations at significantly higher temperatures. In case of reducing conditions, e.g., under Ar/H_2_, in-situ Raman investigations are possible at even higher temperatures. Montagnac et al. recorded the Raman data of pyrolytic graphite (PG) and highly orientated pyrolytic graphite (HOPG) up to temperatures of about 2400 °C [23]. To our knowledge, no other carbon allotropes have been investigated up to such high temperatures, and no investigations higher than 600 °C have been performed in air.

Within the allowed phonon modes of crystalline carbon only the E_2g_ mode (G band) is Raman active. The thermodynamic derivation of its frequency change Δ*ω* with temperature *T*, volume *V* and pressure *p* is described by [12]:

(1) $$\Delta \omega =(\chi_{T}+\chi_{V})\Delta T={\left(\frac{d\omega }{dT}\right)}_{V}\Delta T+{\left(\frac{d\omega }{dV}\right)}_{T},\Delta V={\left(\frac{d\omega }{dT}\right)}_{V}\Delta T+{\left(\frac{d\omega }{dV}\right)}_{T}{\left(\frac{d\omega }{dT}\right)}_{p}\Delta T$$

with χ_T_ the intrinsic temperature effect due to the direct coupling of phonon modes and χ_V_ the volume effect due to the thermal expansion. The thermal expansion of the graphite crystal occurs mainly along the *c*-axis perpendicular to the graphene layers of its hexagonal structure (space group: P6_3_*mc*), resulting in a small effect on the in-plane E_2g_ mode. Hence, Equation (1) can be reduced to:

(2) $$\Delta \omega =\;\chi_{T}\cdot \Delta T$$

According to Equation (2), the G line shifts linearly with temperature, only few works report a non-linear shift [24,25].

It is well known that the temperature dependence of the phonon dispersion curves of carbon materials is a direct consequence of anharmonic contributions to the lattice potential [26]. According to the molecular dynamics simulation (MD) of Koukaras et al., each of the inserted potentials produces a good description of some phonon branches but none works for all of the optical and acoustical branches of graphene [12,27]. However, the modified Tersoff potential [28] correctly describes the temperature dependence of the E_2g_ mode (Raman G band). A linear down-shift of the G line or softening of the E_2g_ mode, respectively, is predicted within the temperature range investigated (−200 to 1200 °C). Since the G band is associated with the in-plane vibration in sp^2^ carbon, the shift with temperature should be similar for the various carbon materials, i.e., we expect a linear dependence for carbon enclosed in the SiOC matrix with a temperature coefficient χ_G_ of about −0.02 cm^−1^/°C, as experimentally observed in graphene [12,29] HOPG [30] and SWNTs [31,32,33] at low temperatures (T < 600 °C).

## 2. Materials and Methods

In order to synthesize polymer-derived SiOC ceramics, commercially available preceramic polymer resins of the companies Starfire Systems Ltd. (Glenville, NY, USA) (Polyramic^®^: SPR 212) and Wacker Chemie AG (München, Germany) (BELSIL^®^ PMS-MK) have been used. The preceramic polymers were thermally crosslinked in an alumina tube furnace at *T* = 250 °C for 2 h and subsequently pyrolyzed at *T* = 1100 °C for 2 h with a heating and cooling rate of 100 °C/h under a constant argon flow (5 L/h). To ensure an oxygen-free atmosphere during calcination, the chamber was evacuated several times and purged with high purity argon. The resulting black glass was milled and sieved to a particle size below ≤40 µm. Subsequently, the powder was sealed within graphite foils and hot-pressed using the spark plasma sintering technology without any sinter additives at *T* = 1600 °C with a uniaxial load of *p* = 50 MPa for 15 min under high purity argon atmosphere and a heating rate of 320 °C/min. After the pressure-assisted sintering process, black dense monoliths were obtained and cut into cylindrical shape (*d* = 5 mm) using a grinding machine equipped with a diamond grinding wheel.

Raman spectra were collected using a Renishaw InVia Raman microscope equipped with an Ar laser (*λ* = 514.5 nm). The measurements were performed by using a grating of 600 g·mm^−1^ and a confocal microscope (magnification 100× NA 0.9) with a 100 µm aperture and a lateral resolution of 2–4 µm. The spectra were recorded in the wavenumber region of 400–3100 cm^−1^ with a resolution of <1 cm^−1^ at RT and <3 cm^−1^ at 1000 °C. The laser power (20 mW) was attenuated by using neutral density filters; thus, the power on the sample was in the range from 6 µW to 2 mW. In-situ heating of the samples was performed using a TS1500 heating stage from Linkam with a heating rate of 100 °C/min up to *T* = 1000 °C.

## 3. Results and Discussion

**Figure 1:**
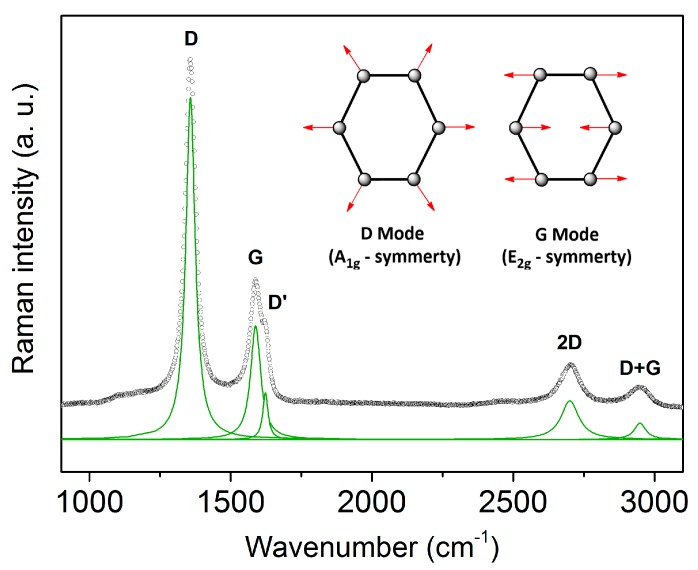
Room-temperature Raman spectrum of C-11. Green line: Lorentzian fits to the Raman modes. The inset shows the vibration modes of the D and G band.

Figure 1 shows a typical room-temperature Raman spectrum of a C-11 sample synthesized at *T* = 1600 °C with the D band at 1350 cm^−1^, the G band at 1590 cm^−1^ and the D’ band at 1620 cm^−1^. The D band is induced by any kind of disorder. It is forbidden in graphite, but becomes Raman active through a disorder-induced double resonance Raman process [34,35], which causes in-plane breathing vibrations of the aromatic ring structures (A_1g_ symmetry). The D’ band is also a disorder-induced double resonance process and is often found in nano-crystalline graphite [36,37]. The G band is assigned to the in-plane stretching vibration of sp^2^ carbon (E_2g_ symmetry). Two additional features are observed at 2700 and 2900 cm^−1^ labeled as the 2D band (an overtone of the D band) and the D + G band (a combination of the D and G band) [6,38].

The presence of both the G and D’ band in the C-11 (and C-17) spectra indicates nano-crystalline carbon. The values of the lateral crystal size (*L_a_*) and of the average distance between lattice defects (*L_D_*) provide further evidence for this kind of carbon. According to Cançado et al.

(3) $$L_{a}=\left(2.4\times 10^{-10}\right)\cdot \lambda_{L}^{4}\cdot {\left(\frac{A_{D}}{A_{G}}\right)}^{-1}$$

with *λ_L_* the wavelength of the laser and *A_D_* and *A_G_* the area of the D and G band [39]. From Equation (3), an average lateral crystal size of *L_a_* ≈ 10 nm can be derived for C-11 and C-17.

**Figure 2:**
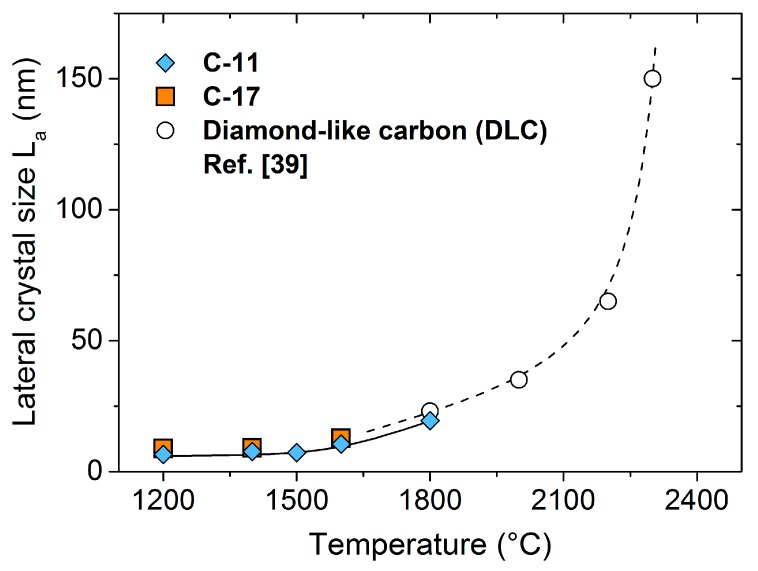
Lateral crystal size *L_a_* of carbon as a function of the temperature. Open points: Adapted from ref. [39], dashed line: guideline for the eye.

The graphitization of diamond-like carbon within the temperature range of 1800–2700 °C has been investigated by Cançado et al. [39]. The lateral crystal size strongly increases from ≈20 nm (1800 °C) to ≈500 nm (2700 °C). As shown in Figure 2, the *L_a_* values for C-11 and C-17, typically about 10 nm within 1200 < *T* < 1600 °C, join at the onset of graphite formation, further supporting the assignment to nano-crystalline carbon.

**Figure 3:**
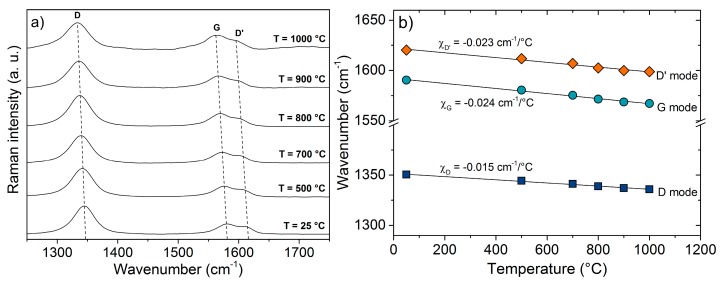
Temperature-dependent Raman spectra (**a**) and thermal coefficients of the Raman bands (**b**) of C-11.

The phonon dispersion curves of nano-crystalline carbon are expected to be different from that of well-ordered carbon (HOPG, graphene) due to the different microstructure and the presence of defects and impurities. Their influence can be followed by the investigation of the thermal shift of the G band of the different carbon structures. Figure 3a presents the Raman spectra of C-11 within the temperature range 25–1000 °C. A linear shift to lower frequencies occurs for all modes G, D and D’.

The shift to a lower wavelength of the G mode with temperature results from an anharmonic contribution to the lattice potential and verifies the results of the MD simulation by Koukaras et al. [27] using the Tersoff potential modified by Lindsay and Broido [28].

The thermal coefficients χ, see Equation (2), are depicted in Figure 3b for the sample C-11. Very similar shifts are seen for C-17. The obtained values for the G band of C-11 (χ_G_ = −0.024 cm^−1^/°C) and C-17 (χ_G_ = −0.023 cm^−1^/°C) are significantly larger than that for well-ordered graphene (χ_G_ = −0.016 cm^−1^/°C) and HOPG (χ_G_ = −0.011 cm^−1^/°C), but smaller than the values for carbon nanowalls (CNWs) [40] and commercial activated graphite [41]: χ_G_ = −0.029 cm^−1^/°C and χ_G_ = −0.027 cm^−1^/°C, respectively. Thus, as in activated graphite, defects and impurities appear to be decisive for the temperature dependence of the Raman G band. Table 1 summarizes the thermal coefficients χ_G_ of the different carbon materials.

The thermal coefficients of the D and D’ band in C-11 and C-17 (χ_D_ = −0.015 and χ_D’_ = −0.023 cm^−1^/°C) match the values observed for CNTs (χ_D_ = −0.014 and χ_D’_ = −0.015–0.020 cm^−1^/°C) [33] containing a significant amount of impurities.

**Figure 4:**
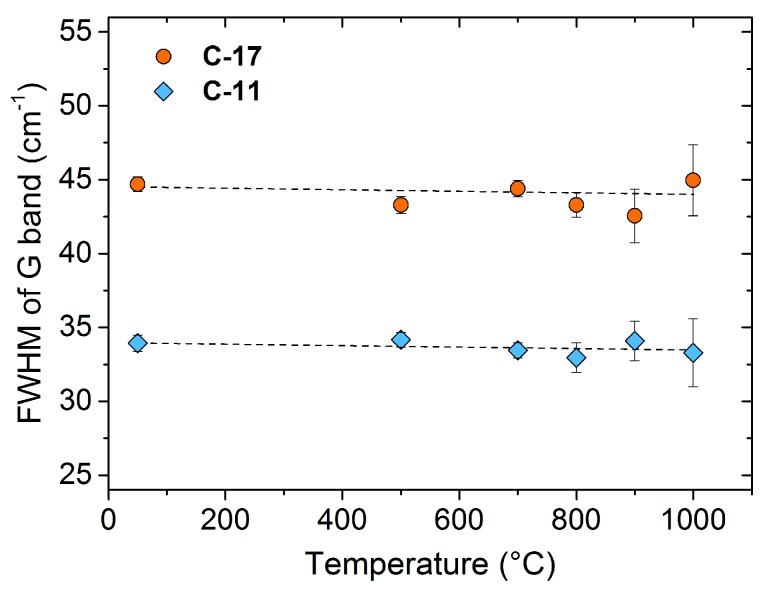
Temperature dependence of the FWHM of the G band of C-11 and C-17.

Apart from the G shift, information about the phonon properties can be obtained from the width of the G line. Figure 4 shows the temperature dependence of the G-line width of C-11 and C-17. The full width at half maximum (FWHM) of both samples is temperature independent, with values of 35 cm^−1^ and 45 cm^−1^. The error bars indicate the standard deviation of the FWHM values. Due to thermal radiation, the error significantly increases with temperature. Theoretical data for graphite and graphene yield a FWHM of ≈12 cm^−1^, which decreases with increasing temperature above *T* > 25 °C due to phonon-phonon interaction [43]. Experimental data for HOPG provide a FWHM of ≈15 cm^−1^ independent of temperature (up to ≈700 °C), [23] suggesting that the phonon-phonon interaction is compensated by phonon-electron interactions (which typically increase with temperature) [23]. In comparison to these well-ordered carbon materials, the significantly larger FWHM of nano-crystalline C-11 and C-17 cannot be solely explained by phonon-phonon and phonon-electron interactions; scattering with defects and impurities most likely determines the line width of the G band in these less-ordered materials.

The G and D’ bands of nano-crystalline carbon in C-11 and C-17 seem to be correlated, since very similar constant ratios of the line widths of the G and D’ bands are observed. The ratio FWHM_G_(C-11)/FWHM_G_(C-17) accounts to 0.77 and FWHM_D’_(C-11)/FWHM_D’_(C-17) is 0.82, respectively. The close correspondence suggests that probably one type of defect in the nano-crystalline structure defines the phonon lifetimes τ (τ ~ FWHM^−1^) of the G and D’ band. C-17 contains carbon and defect concentrations higher than those of C-11. Supposing that scattering with the defects determines the phonon properties in a similar fashion, the FWHM of both lines will increase with the defect concentration. At the same time, the ratio between FWHM and defect concentration remains constant, as indicated by the experiment.

**Figure 5:**
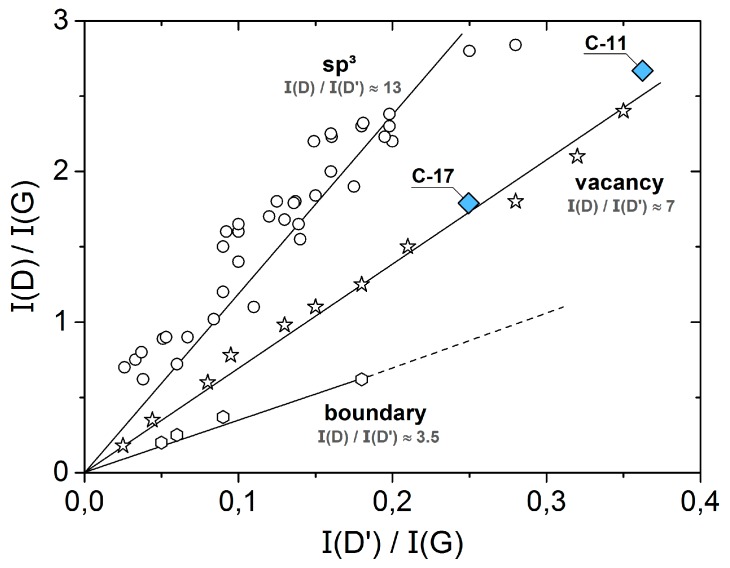
I_D_/I_G_ versus I_D’_/I_G_-plot. Open symbols: ref. [44].

Information about the type of defects in carbon can be derived from the intensity ratios of the D, D’ and G bands. A systematic study of the defects in graphene and graphite has been performed by Eckmann et al. [44]. By plotting the intensity ratio I_D_/I_G_ versus I_D’_/I_G_, different types of defects can be distinguished by the respective slopes (sp^3^: ≈13, vacancies: ≈7 and boundaries: ≈3.5). Figure 5 compares C-11 and C-17 with graphite and modified graphene. C-11 and C-17 fit into the I_D_/I_G_ versus I_D’_/I_G_—dependency observed for vacancies, suggesting that this type of defect dominates the phonon characteristics of nano-crystalline carbon in the silicon oxycarbide ceramics.

## 4. Conclusions

In summary, we report on the Raman data of nano-crystalline carbon in SiOC containing 11 and 17 vol % carbon (samples C-11 and C-17) in the temperature range of 25–1000 °C. The temperature coefficient of the Raman G band is χ_G_ = −0.024 ± 0.001 cm^−1^/°C for both samples, very similar to that of disordered carbon materials, e.g., commercial activated graphite or carbon nanowalls. The observed linear temperature dependence of the G mode up to 1000 °C agrees with the description of the anharmonicity in the lattice potential by the modified Tersoff potential. The line width of the G (and D’) band is temperature independent and significantly larger than that of well-ordered HOPG or graphene indicating that the phonon characteristics are mainly determined by the interaction with defects and impurities. According to the Raman analysis, vacancies are predominant within the nano-crystalline carbon lattice.

## Figures and Tables

**Table 1:** Temperature coefficients of carbon-based materials.

Sample	χ_G_ (cm^−1^/°C)	Temperature Range (°C)	Reference
graphene	−0.016	−200–100	[12]
HOPG	−0.011	10–370	[41]
C-17	−0.023	25–1000	this work
C-11	−0.024	25–1000	this work
activated C	−0.027	150–500	[42]
CNW	−0.029	25–600	[40]
